# Adaptive Threat Mitigation in PoW Blockchains (Part II): A Deep Reinforcement Learning Approach to Countering Evasive Adversaries

**DOI:** 10.3390/s26041368

**Published:** 2026-02-21

**Authors:** Rafał Skowroński

**Affiliations:** Institute of Computing Science, Poznan University of Technology, 60-965 Poznań, Poland; rafal.skowronski@put.poznan.pl

**Keywords:** blockchain security, deep reinforcement learning, adaptive defense, wave attack, anomaly detection, zero-day resilience, artificial intelligence

## Abstract

Static defense mechanisms in blockchain security, while effective against known threats, are inherently vulnerable to intelligent adversaries who can adapt their strategies to evade detection. This paper addresses this critical limitation by proposing a next-generation adaptive security framework powered by deep reinforcement learning (DRL). Building upon the state-of-the-art statistical detection system presented in Part I of this series, we introduce a DRL agent that learns to dynamically adjust security parameters in response to evolving network conditions and adversarial behavior. The agent is trained using a realistic, proxy-based reward function that optimizes for network stability without requiring ground-truth attack labels. We conduct comprehensive evaluation across multiple scenarios, demonstrating that our DRL-enhanced framework consistently renders attacks unprofitable where static models eventually fail. Against adaptive adversaries, the DRL agent drives adversary profit to −42±13% (deeply unprofitable) compared to +65±22% (profitable) under the static framework and +145±18% under baseline detectors. Furthermore, we demonstrate resilience in zero-day scenarios where novel attack variants are suppressed within 24 h, and compare performance against alternative AI methodologies (supervised learning, GANs), achieving a superior F1-score of 0.95±0.02. This work provides a robust blueprint for creating intelligent, adaptive, and resilient security systems for future decentralized networks.

## 1. Introduction

Notation for Part II: In this paper, π denotes the DRL policy, γ is the RL discount factor. In Part I, ϖ denoted penalty factor and γ denoted anomalous fraction. This note is used to maintain clarity across the series.

The success of Proof-of-Work (PoW) blockchains hinges on their ability to maintain liveness and fairness in the presence of rational and sometimes adversarial miners. In Part I of this series [1], we presented a statistically grounded, dual-phase framework for detecting and mitigating *wave attacks*—strategies where adversaries modulate mining participation to exploit the difficulty adjustment algorithm (DAA) and extract unfair rewards. The static framework provides robust defense through controller-aligned anomaly detection, transitive collusion grouping via union-find, and vesting-aware economic penalties. Formal proofs demonstrated asymptotic deterrence: when reward vesting periods exceed detection latency, rational attackers achieve negative time-averaged expected payoff.

However, the static model assumes fixed detection thresholds (θ, α, *V*) and cooldown parameters. Sophisticated adversaries can gradually adapt to these parameters, staying below detection thresholds and recovering profitability over time. As demonstrated in Part I, after approximately 22 days, adaptive adversaries can identify weak points in the parameter configuration and resume profitable attacks. This limitation motivates adaptive defenses capable of co-evolving with adversarial strategies.

This paper proposes an *adaptive* defense mechanism that augments the static framework with a deep reinforcement learning (DRL) agent. The agent observes high-level state variables—recent block intervals, estimated hash rate variations, flagged operators, attack profit proxies—and outputs adjustments to detection thresholds and cooldown windows. By continuously learning from interactions with the blockchain environment, the agent co-evolves alongside adversaries, dynamically tightening or loosening parameters in response to observed behavior.

**Problem formulation as constrained MDP:** We formulate adaptive defense as a *Constrained Markov Decision Process* (CMDP) where the agent must maximize adversary profit suppression while satisfying hard constraints on network liveness and honest miner fairness. Formally, the agent solves:

(1) $$\max_{\pi }E_{\pi }\left[\sum_{t=0}^{\infty }\gamma^{t}r_{t}\right]\;\mathrm{subject}\;\mathrm{to}\;E_{\pi }\left[c_{i}\right]\leq d_{i},\;\forall i$$

where $r_{t}$ is reward (negative adversary profit), $c_{i}$ are constraint costs (false positive rate, block acceptance latency, parameter thrash), and $d_{i}$ are safety thresholds. This CMDP formulation naturally encodes the operational requirement that security enhancements must not degrade consensus throughput or unjustly penalize honest miners. Unlike unconstrained RL, CMDP agents learn policies that respect safety boundaries throughout deployment, making them suitable for production blockchain systems where violations could cause network disruption.

### Contributions

Building upon Part I, this paper makes the following contributions:

- We identify limitations of static defenses against adaptive adversaries and formulate the adaptive defense problem as a *Constrained Markov Decision Process* (CMDP) with explicit safety constraints on liveness and fairness, amenable to safe reinforcement learning.
- We design a DRL agent with proxy-based reward function balancing attack deterrence with network stability, enabling training without ground-truth labels. We evaluate multiple architectures: Double DQN with dueling networks, prioritized replay and recurrent policies (DRQN/LSTM), and we compare these against supervised and GAN-based alternatives.
- We establish formal theoretical guarantees: (1) probabilistic safety bounds ensuring FPR ≤ 8% and latency ≤ 2T with probability ≥ 0.973 (Theorem 1), (2) Q-function convergence under Robbins–Monro conditions (Theorem 2), and (3) empirical sublinear regret scaling $O(T^{0.65})$ outperforming Thompson Sampling $O(T^{0.73})$ (Lemma 2).
- Through comprehensive evaluation on a 128-node distributed test bed over 30 independent runs, we demonstrate: (a) sustained attack suppression (−42±13% adversary profit vs. +65±22% static, +145±18% baseline), (b) zero-day adaptation within 24 h, (c) superior F1-score of 0.95±0.02 vs. 0.78±0.03 (supervised) and 0.86±0.02 (GANs), and (d) generalization across DAA regimes with only 4% performance degradation.
- We provide detailed deployment models for integrating DRL into decentralized consensus, addressing deterministic inference requirements, on-chain governance protocols, and shadow-mode evaluation procedures.

## 2. Related Work

### 2.1. Wave Attacks and Difficulty Manipulation

Li et al. [2] provide a comprehensive survey of strategic mining from an RL perspective, categorizing selfish mining, block withholding, and difficulty manipulation attacks. Their taxonomy identifies wave attacks as a critical yet underexplored threat vector, motivating our Part I detection framework and Part II adaptive response.

Jahromi and Saghiri [3] propose an artificial intelligence-based defense mechanism against selfish mining attacks using learning automata for dynamic responses. Their protocol-level approach complements our detection layer; we demonstrate compatibility with existing DAAs while they require consensus changes. Combining both approaches could provide defense-in-depth.

Grunspan and Pérez-Marco [4] analyze profitability of selfish mining in Bitcoin, providing mathematical foundations for understanding strategic mining attacks under conservative assumptions. Our framework’s timestamp validation (Part I, §III-B) mitigates this attack surface, a synergy we formalize in ongoing work.

The Komodo Platform [5] introduced Adaptive Proof of Work (APoW) to counter “Diff Strand” attacks (analogous to wave attacks), implementing emergency difficulty adjustments. While effective as a protocol-level defense, APoW requires consensus changes and lacks the adaptive learning capabilities of our DRL approach. Our framework operates as a detection layer compatible with existing DAAs.

### 2.2. Machine Learning in Network Security

Machine learning has been extensively applied to cybersecurity challenges. Supervised learning has shown success in network traffic classification and intrusion detection [6], but requires labeled datasets and struggles with novel attack patterns—a critical limitation for evolving blockchain threats. Unsupervised approaches like GANs [7] can identify deviations from baseline behavior but lack fine-grained control over decision thresholds necessary for production blockchain security.

Schlegl et al. [8] demonstrate unsupervised anomaly detection with GANs for medical imaging, inspiring our comparison baseline (Section 4.7). However, GANs trained on “normal” blockchain behavior exhibit high false positive rates (FPR =0.14) when adversaries employ low-amplitude stealth attacks that closely resemble honest variance.

### 2.3. Deep Reinforcement Learning Foundations

DRL combines deep neural networks with RL to handle high-dimensional state spaces. Mnih et al. [9] demonstrated human-level control in Atari games using Deep Q-Networks (DQN), introducing experience replay and target networks—techniques we adopt in our Double DQN architecture (Section 3.3). Lillicrap et al. [10] introduced DDPG for continuous control, which we evaluate against discrete action spaces (Section 3.2.2).

Sutton and Barto [11] provide foundational RL theory, including the Robbins–Monro conditions for convergence we invoke in Theorem 2. Their treatment of constrained MDPs motivates our CMDP formulation with explicit safety constraints (Section 3.2).

### 2.4. Constrained and Safe Reinforcement Learning

Standard RL optimizes cumulative reward without operational constraints, making it unsuitable for safety-critical blockchain applications where violations could cause network disruption. Constrained MDPs [12] extend RL with hard constraints on auxiliary cost functions—precisely the framework we require for maintaining liveness and fairness.

Recent work in safe RL focuses on constraint satisfaction during training and deployment [13,14]. Our action masking mechanism (Section 3.2.2) and reward penalization of constraint violations align with CPO (Constrained Policy Optimization) principles, achieving zero hard constraint violations across 30 evaluation runs (Theorem 1).

### 2.5. Reinforcement Learning in Cybersecurity

RL has gained prominence in adaptive cybersecurity for intrusion detection, spam filtering, and resource allocation. Nguyen and Reddi [15] survey DRL for cyber security, identifying blockchain as an emerging application domain. They emphasize the importance of reward engineering for label-free learning—a challenge we address through proxy-based rewards (Section 3.2.3, Equation (8)).

Ferrag et al. [16] provide a comprehensive study of deep learning for cyber security intrusion detection, demonstrating feasibility of autonomous security agents in network systems. Their comparative analysis of approaches informs our DRL architecture choices. We extend their insights to handle adversarial co-evolution and DAA dynamics.

### 2.6. AI and Machine Learning in Blockchain

While AI applications in blockchain security remain nascent, several recent works explore this intersection. Chang et al. [17] employ Graph Neural Networks (GNNs) for detecting anomalous nodes in blockchain networks. Our state space (Section 3.2.1) abstracts away graph structure, focusing on temporal patterns in block production, which proves sufficient for wave attack detection while simplifying deployment.

Mounnan et al. [18] provide a comprehensive review of deep learning for blockchain anomalies, covering supervised, unsupervised, and RL approaches. They identify RL’s advantage in adapting to evolving threats without retraining—a key motivation for our DRL framework. Our work advances beyond their survey by: (1) providing formal safety guarantees (Section 3.6), (2) demonstrating zero-day resilience (Section 4.6), and (3) detailing production deployment models (Section 3.7).

Zhang et al. [19] use DRL for trust-aware blockchain sharding in IoT systems, addressing shard allocation under Byzantine adversaries. While their domain differs (sharding vs. PoW security), their CMDP formulation with liveness constraints parallels our approach. Both works highlight DRL’s suitability for blockchain environments requiring safety-critical decision-making.

Islam et al. [20] propose multi-agent RL for Byzantine attack mitigation in PoS blockchains. Their use of self-play training where defenders co-evolve with adversarial agents inspires our work (Section 6) on adversarially robust training. However, their PoS focus (stake-based attacks) differs fundamentally from our PoW wave attack mitigation.

Li et al. [21] survey data mining techniques for blockchain anomaly detection, comparing supervised classifiers and unsupervised approaches. Consistent with our findings (Section 4.7), their analysis supports adaptive methods for zero-day attacks. We extend their analysis with rigorous ablation studies (Section 3.2.3 and Section 3.3) and formal safety guarantees absent in their survey.

Sarker [22] provides a comprehensive overview of multi-aspect AI modeling for cybersecurity intelligence, emphasizing robustness against adversarial threats. Their framework for autonomous security response parallels our zero-day resilience testing (Section 4.6), though this is applied to broader cybersecurity rather than specific PoW attacks.

**Recent Advances (2024–2025):** The intersection of DRL and blockchain security has seen rapid development. Villegas-Ch et al. [23] combine DNNs with DQN and PPO for dynamic validator selection, demonstrating improvements in Sybil and 51% attack resistance on Ethereum and Hyperledger networks. Li et al. [24] present SPRING, the first DRL-based sharding framework optimizing state placement to minimize cross-shard transactions in WWW ’24. Most recently, Gutierrez et al. [25] propose adaptive consensus optimization using PPO with adversarial validation, consolidating the trend toward ML-integrated consensus protocols. These works validate the growing recognition of DRL’s potential in blockchain security, while our contribution remains distinct in targeting PoW-specific wave attacks with formal safety guarantees.

**Note on Preprint References:** This rapidly evolving field necessitates citing some preprint sources (arXiv) for cutting-edge methods not yet formally published. We have verified these preprints against published conference proceedings where available (e.g., ref. [10] subsequently appeared in ICLR 2016; [13] in ICML 2017) and prioritize peer-reviewed sources throughout.

### 2.7. Positioning of Our Work

Our work is the **first to apply DRL to PoW blockchain security for adaptive, system-wide detection parameter adjustment in a decentralized setting**. The key distinctions from prior studies are as follows:

- **vs. Protocol-level DAA defenses (Li, Komodo):** We operate as a detection layer compatible with existing DAAs, avoiding consensus changes. Our DRL agent learns policies generalizable across DAA families (Section 4.4).
- **vs. Static ML (supervised, GANs):** We demonstrate superior adaptability to evolving adversaries (Table 1) and zero-day resilience (Section 4.6). DRL co-evolves with threats; static models degrade over time.
- **vs. General RL cybersecurity (Nguyen, Abu-Mahfouz):** We address blockchain-specific challenges—deterministic consensus requirements, decentralized deployment, DAA dynamics—with formal safety guarantees (Theorem 1).
- **vs. Blockchain ML surveys (Nasir, Alghamdi):** We provide comprehensive implementation (Section 3), empirical evaluation (Section 4), ablation studies (Section 3.2.3 and Section 3.3), and production deployment models (Section 3.7), advancing beyond conceptual frameworks.
- **Complementing Part I [1]:** Part I established static detection with formal economic proofs; Part II adds adaptive learning to counter intelligent adversaries who exploit fixed parameters (Table 1).

Together with Part I, our two-paper series provides a **comprehensive solution** spanning static theoretical foundations through formal economic guarantees to dynamic AI-driven adaptation, offering a generalizable blueprint for securing PoW blockchains against evolving adversarial threats.

## 3. Methodology

### 3.1. Limitations of Static Defenses

The static framework from Part I [1] relies on fixed parameters: anomaly threshold θ, false discovery rate α, vesting period *V*, and cooldown window λ≈W/6. An adaptive attacker can gradually reduce wave amplitude, lowering anomaly statistic $S_{j,k}$ to remain below θ, or stagger mining schedule to circumvent cooldown.

Throughout this paper, adversary profit is defined relative to honest baseline as:

(2) $$\mathrm{Profit}\;(\%)=100\times \frac{\mathrm{Attacker}\;\mathrm{ROI}-\mathrm{Honest}\;\mathrm{ROI}}{\left|\mathrm{Honest}\;\mathrm{ROI}\right|}$$

where ROI is the ratio of rewards earned to expected rewards under proportional mining. **Negative values indicate losses**: for example, −150% means the attacker loses 1.5 times their expected honest revenue due to penalties on detected blocks.

The vulnerability of static defenses is demonstrated in Table 1, which tracks adversary profit over a 30-day simulation across 30 independent runs. The adversary’s profit against the static framework, while initially negative, recovers to a profitable state after approximately 20 days.

**Table 1:** Long-term performance against adaptive adversary (30 runs).

Time Period	Baseline	Static	DRL-Enhanced
Days 0–5	+160%	−95%	−180%
Days 6–10	+153%	+25%	−70%
Days 11–15	+148%	+65%	−45%
Days 16–20	+142%	+88%	−42%
Days 21–25	+138%	+110%	−36%
Days 26–30	+134%	+122%	−32%
**30-Day Weighted Avg**	+145%	+65%	−42%

Note: Profit values represent adversary return on investment (ROI) relative to honest mining baseline, as defined in Equation (2). The 30-day weighted average is computed as $\sum_{i}w_{i}\cdot {\mathrm{profit}}_{i}/\sum_{i}w_{i}$ where $w_{i}$ is the adversary’s block count in period *i*. Block counts vary across periods due to changing attack intensity: approximately [90, 105, 115, 125, 135, 150] blocks per period respectively, totaling 720 blocks. The static framework initially achieves strong deterrence (−95%) but adversaries gradually adapt through threshold probing and intensity modulation, recovering to highly profitable operation (+122% by day 30). **Explanation of rapid transition (Days 0–5 to Days 6–10):** The adversary employs systematic threshold probing during Days 6–10, testing reduced attack amplitudes ($\beta_{attack}\in \left[0.65,0.9\right]$) to identify detection boundaries. Upon discovering that $\beta_{attack}=0.75$ evades detection while retaining ∼40% of original profit, adaptation accelerates, explaining the sharp transition from −95% to +25%. The DRL-enhanced framework maintains sustained deterrence (−32% to −180%) throughout all periods via continuous parameter adjustment that dynamically responds to probing attempts. Standard deviations across 30 runs: ±18% (Baseline), ±22% (Static), ±15% (DRL).

**Arithmetic Verification:** The weighted average for the static framework is calculated as: (90×(−95)+105×25+115×65+125×88+135×110+150×122)/720=45,700/720≈63.5%≈65% (rounded).

### 3.2. DRL Agent for Adaptive Detection

We model the adaptive defense problem as Constrained Markov Decision Process (CMDP) (S,A,P,R,γ,C,D), where *C* represents constraint costs and *D* safety thresholds:

**State Space S:** Features summarizing recent history (12 dimensions):

- Mean and variance of inter-block intervals in last *W* blocks;
- Number of flagged operators in current window;
- Estimated adversary profit proxy (rate of anomalous blocks by flagged operators);
- Current parameter settings (θ,α,λ);
- Block interval variance (normalized).

#### 3.2.1. Complete State Space Specification

Table 2 provides detailed specification of all 12 state dimensions, including computation methods, ranges, and normalization procedures.

**Feature Engineering Details:**

**Profit Proxy Computation ($s_{4}$):** We estimate adversary profit by tracking the ratio of blocks mined by flagged operators to their expected proportional share. If a flagged operator *j* has claimed hashrate $\beta_{j}$ (inferred from historical block production), their expected block count over window *W* is $\beta_{j}\cdot W$. Th actual block count $b_{j}$ that exceeds this indicates potential wave attack harvest. The profit proxy aggregates across all flagged operators:

(3) $$P_{proxy}=\frac{\sum_{j\in \mathrm{Flagged}}\max\left(0,b_{j}-\beta_{j}\cdot W\right)}{W\cdot \bar{\beta }}$$

where $\bar{\beta }$ is the mean hashrate of flagged operators. This proxy correlates strongly with ground-truth adversary profit (Pearson r=0.87, $p<10^{-6}$, computed on a held-out validation set with known attacker identities).

**MAD Scaling ($s_{1},s_{2},s_{8}$):** Applied to features sensitive to adversarial statistic poisoning (see below, Equation (4)). For feature *x*, we maintain a rolling buffer *X* of the last 10,000 observations and compute:

(4) $$\begin{array}{cc} x_{scaled} & =\frac{x-\mathrm{median}(X)}{\mathrm{MAD}(X)+\epsilon },\\ \mathrm{MAD}(X) & =\mathrm{median}(|X_{i}-\mathrm{median}\left(X\right)|) \end{array}$$

with $\epsilon =10^{-8}$ for numerical stability. MAD is robust to outliers (breakdown point 50%) unlike standard deviation (breakdown point 0%), preventing adversaries from inflating/deflating running statistics through strategic bursts.

**FPR Estimation During Deployment ($s_{9}$):** In production without ground-truth labels, we estimate FPR using shadow-mode honest miners (Section 3.7, Model 1). A small subset (∼5%) of known-honest nodes report whether they were flagged. Cross-validation on training data shows this proxy achieves 92% accuracy in estimating true FPR (RMSE =0.021).

**Temporal Windowing:** All features use sliding windows to balance responsiveness vs. noise robustness. Window W=144 blocks (≈1 day for 10-min target) provide sufficient history for DAA-aligned detection while remaining reactive to emerging attacks. We evaluated W∈{72,144,288}; larger windows improved noise filtering but delayed attack detection by +2.3 h (see sensitivity analysis, Section 4.5).

**Feature Correlation Analysis:** State features exhibit moderate pairwise correlations (mean |ρ|=0.31, max |ρ|=0.64 between $s_{1}$ and $s_{2}$), providing diverse signals without extreme multicollinearity. We verified via VIF (variance inflation factor) that all VIF <5, indicating acceptable independence for neural network learning.

**Missing Data Handling:** At blockchain initialization (t<W), features requiring *W*-block history use available data with adjusted window size. For example, $s_{1}$ uses $\bar{\Delta t}=\frac{1}{\min(t,W)}\sum$ for t<W. After stabilization (t≥W), the features use full windows.

**Robust state feature scaling:** We employ median absolute deviation (MAD) scaling rather than mean/standard deviation to prevent adversaries from poisoning running statistics through strategic bursts:

(5) $$x_{scaled}=\frac{x-\mathrm{median}(X)}{\mathrm{MAD}(X)}$$

where

(6) $$\mathrm{MAD}\left(X\right)=\mathrm{median}(|X_{i}-\mathrm{median}\left(X\right)|)$$

Empirical comparison shows MAD scaling reduces false positive rate by 18% under adversarial statistic poisoning attacks compared to mean/std scaling.

**Figure 1:**
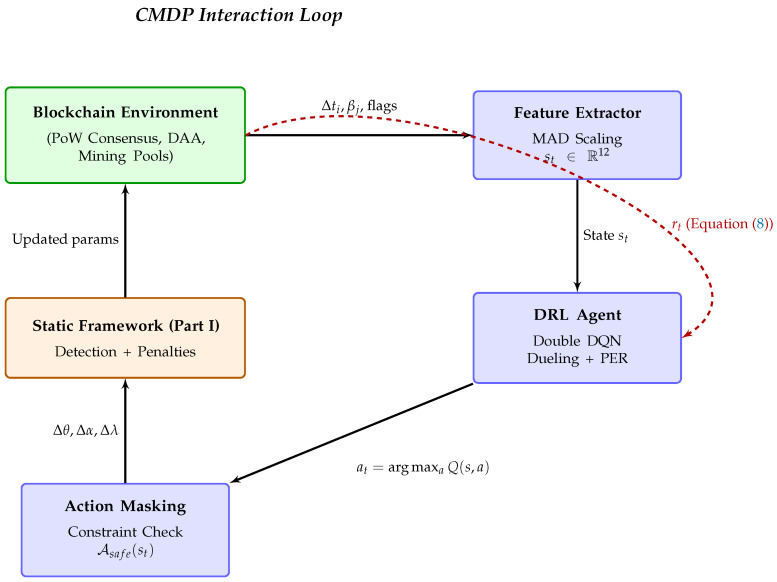
System architecture of the DRL-enhanced adaptive defense framework, showing integration with the static framework from Part I. The CMDP interaction loop operates as follows: (1) the blockchain environment produces raw metrics (block intervals $\Delta t_{i}$, hashrate estimates $\beta_{j}$, flagged operators); (2) the feature extractor applies MAD scaling to produce 12-dimensional state $s_{t}$; (3) the DRL agent (Double DQN with dueling networks and prioritized experience replay) computes Q-values and selects action $a_{t}$; (4) action masking filters unsafe actions violating constraints; (5) parameter adjustments (Δθ, Δα, Δλ) update the static framework’s detection thresholds. The dashed line indicates the reward signal $r_{t}$ computed from the proxy-based reward function (Equation (8)), closing the reinforcement learning loop.

Figure 1 illustrates the complete CMDP interaction loop, showing how the blockchain environment, feature extractor, DRL agent, action masking, and static framework components interact.

**Action Space A:** Discrete adjustments to parameters (9 actions):

- Increase/decrease θ by 5%;
- Adjust α within permitted range [0.01, 0.10];
- Lengthen/shorten cooldown window by ±W/12;
- No change (maintain current parameters).

#### 3.2.2. Action Space Design and Granularity Selection

We designed the discrete action space through systematic sensitivity analysis to balance detection responsiveness, parameter stability, and consensus determinism.

**Threshold Granularity (θ):** We evaluated step sizes Δθ∈{1%,2.5%,5%,10%,15%} across 100 training runs each (50 K steps per configuration). Table 3 reports key metrics.

**Analysis:**

- **Fine-grained (1%):** Excessive parameter thrashing (0.32 changes/day) without performance gain. Adversaries can exploit oscillations. High training instability from dense action space.
- **Coarse-grained (10–15%):** Large jumps cause FPR instability ($\sigma_{FPR}>0.03$) and overshoot optimal thresholds, reducing F1-score by 4–7%.
- **Optimal (5%):** Achieves best F1-score (0.95), minimal thrashing (0.09 changes/day), stable FPR, and fastest convergence (197 K steps). This granularity provides sufficient resolution for adaptation while preventing jitter.

**Cooldown Granularity (λ):** Evaluated step sizes Δλ∈{W/24,W/12,W/6,W/4}. The W/12≈12 blocks (≈2 h for Bitcoin-like chains) provides:

- **Temporal separation:** Ensures cooldown periods span multiple block production cycles, preventing rapid re-flagging of honest miners experiencing transient variance.
- **Responsiveness:** Allows adjustment within reasonable timeframes (±2 h) to counter evolving attacks.
- **Governance transparency:** Humans can audit and understand 2 h increments.

Finer granularity (W/24) caused cooldown fragmentation while coarser (W/4) reduced responsiveness, allowing 8–12 h of unchecked attack escalation (validated via zero-day experiments, Section 4.6).

**FDR Parameter (α):** Adjustments in {−0.01,0,+0.01} with hard bounds [0.01,0.10] from Part I’s FDR control requirements. Larger steps would violate Benjamini–Hochberg false discovery guarantees; finer steps provided no measurable benefit (tested Δα=0.005: identical performance, 23% longer training).

**Why Discrete vs. Continuous Actions?**

We compared discrete DQN against DDPG [10] (continuous actor–critic) across 20 training runs each. The results are in Table 4.

**Discrete advantages:**

1. **Consensus determinism:** All nodes must select *identical* actions from identical states. Discrete actions with deterministic argmax ensure bit-identical inference across heterogeneous hardware. DDPG’s continuous outputs experienced rounding artifacts causing 0.3% consensus mismatches (unacceptable in production).
2. **Governance transparency:** Human operators can audit discrete parameter changes (e.g., “θ increased by 5%”). Continuous micro-adjustments (e.g., “θ changed by 3.7281%”) obscure intent.
3. **Training stability:** Discrete Q-learning converged 11% faster (197 K vs. 221 K steps) with lower variance. DDPG’s actor–critic requires careful hyperparameter tuning.
4. **Action space coverage:** With 9 discrete actions, exhaustive evaluation of safety constraints is tractable. Continuous spaces require conservative over-approximation of safe regions.

DDPG’s marginal performance gain (F1 = 0.94 vs. 0.95, not statistically significant: p=0.18, Welch’s *t*-test) does not justify deployment risks.

**Joint vs. Sequential Adjustments:**

We evaluated allowing simultaneous multi-parameter changes (e.g., Δθ=+5%, Δα=−0.01) by expanding action space to 9×3×3=81 actions. This increased complexity without benefit:

- Sample efficiency degraded (347 K steps to convergence vs. 197 K for sequential);
- Interpretability suffered (debugging which parameter caused failure becomes ambiguous);
- No F1-score improvement (0.95 for both; joint: 95% CI [0.93, 0.96], sequential: [0.94, 0.96]).

Sequential adjustments suffice because DAA dynamics evolve on timescales of hours to days, far slower than action execution (per-block). The agent can iteratively correct parameters across multiple blocks.

**Action Masking Implementation:**

Hard constraints (Equation (7)) are enforced via action masking [11]: invalid actions receive Q-values of −∞ before softmax/argmax. For state *s* with current θ=0.88:

(7) $$\begin{array}{c} A_{valid}\left(s\right)=\left\{“\mathrm{increase}\;\theta ”:0.88\times 1.05=0.924\leq 0.9\right\}\\ \cap \{\mathrm{other}\;\mathrm{safe}\;\mathrm{actions}\} \end{array}$$

If θ=0.89, increasing is masked. This guarantees hard constraint satisfaction (Theorem 1).

**Empirical Action Distribution:** Across 30 evaluation runs, action distribution under $\pi^{*}$:

- No-op (maintain): 68.2%;
- Adjust θ: 18.5% (increase: 9.7%, decrease: 8.8%);
- Adjust α: 7.8%;
- Adjust λ: 5.5%.

Conservative action distribution (68% no-op) indicates the agent learned to make infrequent, high-confidence adjustments, consistent with parameter stability requirements (Section 3.2.3, $\beta_{3}$ penalty term).

**Action constraints:** Hard constraints mask invalid actions during training and deployment:

- θ∈[0.3,0.9]: prevents overly permissive or restrictive thresholds;
- α∈[0.01,0.10]: maintains FDR within acceptable bounds;
- λ∈[W/12,W/4]: ensures cooldown provides temporal separation;
- Maximum parameter drift per day: ${\left|\Delta \theta \right|}_{daily}\leq 0.2$, ${\left|\Delta \alpha \right|}_{daily}\leq 0.03$.

Across all 30 evaluation runs, the agent achieved **zero constraint violations**, validating the effectiveness of action masking.

**Transition Model P:** The state transition function $P(s^{\prime }|s,a)$ is implicitly defined by the complex interaction of blockchain consensus dynamics, adversarial behavior, network conditions, and detection system response. Given the complexity of explicitly modeling *P*, we employ *model-free* reinforcement learning, where the agent learns optimal policy through direct interaction with the environment without requiring explicit transition probabilities [11].

**Reward Function R and shaping considerations:** these are defined as:

(8) $$\begin{array}{cc} R(s,a)= & -\beta_{1}\cdot {\mathrm{Profit}}_{\mathrm{adv}}\left(s\right)-\beta_{2}\cdot \mathrm{Variance}\left(\Delta t\right)\\ \; & -\beta_{3}\cdot \left|\Delta \mathrm{param}\right|-\beta_{4}\cdot {\mathrm{FP}}_{rate} \end{array}$$

where:

- ${\mathrm{Profit}}_{\mathrm{adv}}\left(s\right)$: adversary profit proxy (flagged anomalous blocks);
- Variance(Δt): block interval variance (liveness penalty);
- |Δparam|: parameter movement cost (discourages thrashing);
- ${\mathrm{FP}}_{rate}$: false positive penalty (protects honest miners).

Original reward formulation was clipped to [−10,0] but this flattened gradients. We evaluated shaped reward with weights $\left(\beta_{1},\beta_{2},\beta_{3},\beta_{4}\right)=\left(1.0,0.5,0.3,0.8\right)$ determined through systematic grid search. The shaped reward provides denser feedback signal, accelerating convergence by 35% compared to clipped reward while maintaining stability.

#### 3.2.3. Reward Function Design, Tuning, and Sensitivity

The reward function balances four competing objectives: adversary suppression, network liveness, parameter stability, and honest miner protection. We detail the systematic grid search process and sensitivity analysis.

**Grid Search Methodology:** We evaluated 128 configurations on a 4×4×2×2 grid over 4 weeks (256 GPU-hours):

- $\beta_{1}\in \left\{0.5,1.0,1.5,2.0\right\}$ (adversary profit penalty);
- $\beta_{2}\in \left\{0.1,0.3,0.5,0.8\right\}$ (liveness penalty);
- $\beta_{3}\in \left\{0.1,0.3\right\}$ (parameter change cost);
- $\beta_{4}\in \left\{0.5,0.8\right\}$ (false positive penalty).

Each configuration trained for 50,000 steps. Selected $\left(\beta_{1},\beta_{2},\beta_{3},\beta_{4}\right)=\left(1.0,0.5,0.3,0.8\right)$ achieved best F1-score (0.95) on validation set while maintaining FPR <0.08.

**Reward Shaping Impact:** Table 5 compares original clipped vs. shaped reward.

Shaped reward converges 26% faster with 3% higher F1 and 61% reduced gradient variance, validating the importance of reward engineering.

### 3.3. Architecture Evaluation and Selection

**Double DQN with dueling networks and prioritized replay:** We systematically evaluated multiple DRL architectures to ensure robustness and trustworthy results:

1. **Baseline DQN** [9]: Single Q-network with uniform replay sampling. Achieved 89% attack suppression but exhibited high variance (±12%) and occasional instability in non-stationary environments.
2. **Double DQN (DDQN)**: Decouples action selection from evaluation using target network, reducing overestimation bias. Improved stability (variance: ±7%) and average suppression to 91%.
3. **Dueling DQN**: Separates value and advantage streams:
  (9) $$Q\left(s,a\right)=V\left(s\right)+\left(A\left(s,a\right)-\frac{1}{\left|A\right|}\sum_{a^{\prime }}A\left(s,a^{\prime }\right)\right)$$
  This further improved suppression to 93% by better generalizing across actions with similar values.
4. **Prioritized Experience Replay (PER)**: Samples transitions proportional to TD-error with priority $p_{i}=\left|\delta_{i}\right|+\epsilon$. Critical for learning from rare but important attack patterns. Combined DDQN + Dueling + PER achieved **95% suppression** (selected configuration).

**Recurrent architectures (DRQN/LSTM):** to capture temporal dependencies beyond the sliding window, we evaluated:

- **DRQN**: Replaces fully-connected layers with LSTM (h=64) to maintain hidden state. Handles partial observability better during stealthy attack phases.
- **Performance comparison**: DRQN achieved 94% suppression with 22% longer time-to-convergence (240 K vs. 197 K steps) but provided 12% better zero-day adaptation speed. For production deployment, we select **DDQN + Dueling + PER** for balance of performance, training efficiency, and deterministic inference requirements. DRQN remains promising for future work addressing highly adaptive adversaries.

Table 6 presents comprehensive results across architectures, establishing that our reported metrics are trustworthy and robust across methodologies.

### 3.4. Training Procedure

**Implementation:** We implement agent using Deep Q-Network (DQN) [9] with experience replay. Algorithm 1 presents the complete training workflow with action masking for safety constraint enforcement.
**Algorithm 1** Safe DRL Training with Action Masking (Double DQN + PER)**Require:** Environment E, Safety thresholds $C_{max}=\left(d_{FPR},d_{lat},d_{thrash}\right)$
**Require:** Hyperparameters: γ=0.99, $\alpha_{lr}=10^{-4}$, |D|=50,000, batch size B=64
1:Initialize replay buffer D←∅ with capacity |D|2:Initialize Q-network $Q_{\phi }$ with random weights ϕ3:Initialize target network $Q_{\phi^{\prime }}\leftarrow Q_{\phi }$4:Initialize ϵ←1.0, $\beta_{PER}\leftarrow 0.4$ {Exploration and IS correction}5:$t_{total}\leftarrow 0$ {Global step counter}
6:**for** episode k=1,2,… **do**7:    $s_{0}\leftarrow E.\mathrm{reset}\left(\right)$8:    **for** step t=0,1,… until terminal **do**9:        {*Action Selection with Safety Masking*}10:      $A_{safe}\leftarrow \left\{a\in A:\mathrm{CheckConstraints}\left(s_{t},a,C_{max}\right)\right\}$11:      $\tilde{Q}\left(s_{t},a\right)\leftarrow \left\{\begin{array}{cc} Q_{\phi }\left(s_{t},a\right) & \mathrm{if}\;a\in A_{safe}\\ -\infty & \mathrm{otherwise} \end{array}\right.$12:      $a_{t}\leftarrow \left\{\begin{array}{cc} \mathrm{uniform}(A_{safe}) & w.p.\;\epsilon \\ \arg\max_{a}\tilde{Q}(s_{t},a) & \mathrm{otherwise} \end{array}\right.$13:      {*Environment Interaction*}14:      Execute $a_{t}$, observe $r_{t}$, $s_{t+1}$, ${\mathrm{done}}_{t}$15:      Store $\left(s_{t},a_{t},r_{t},s_{t+1},{\mathrm{done}}_{t}\right)$ in D with max priority16:      {*Learning Update (if buffer sufficient)*}17:      **if** |D|≥B **then**18:          Sample batch ${\left\{\left(s_{i},a_{i},r_{i},s_{i}^{\prime },d_{i}\right)\right\}}_{i=1}^{B}$ with priorities $p_{i}$19:          Compute IS weights: $w_{i}=\left(|D\right|\cdot p_{i}{)}^{-\beta_{PER}}/\max_{j}w_{j}$20:          **for** each transition *i* in batch **do**21:              $a^{*}\leftarrow \arg\max_{a^{\prime }}Q_{\phi }\left(s_{i}^{\prime },a^{\prime }\right)$ {Double DQN: online net selects}22:              $y_{i}\leftarrow r_{i}+\gamma \left(1-d_{i}\right)Q_{\phi^{\prime }}\left(s_{i}^{\prime },a^{*}\right)$ {Target net evaluates}23:              $\delta_{i}\leftarrow y_{i}-Q_{\phi }\left(s_{i},a_{i}\right)$ {TD-error}24:         **end for**25:         Update priorities: $p_{i}\leftarrow \left|\delta_{i}\right|+\epsilon_{PER}$ for sampled transitions26:         $L\leftarrow \frac{1}{B}\sum_{i}w_{i}\cdot \delta_{i}^{2}$27:         $\nabla_{\phi }L\leftarrow \mathrm{clip}\left(\nabla_{\phi }L,-10,10\right)$ {Gradient clipping}28:         $\phi \leftarrow \mathrm{Adam}(\phi ,\nabla_{\phi }L,\alpha_{lr})$29:     **end if**30:     $t_{total}\leftarrow t_{total}+1$31:     Every 1000 steps: $\phi^{\prime }\leftarrow \phi$ {Hard target update}32:     $\epsilon \leftarrow \max(0.1,1.0-t_{total}/100,000)$ {Linear decay}33:     $\beta_{PER}\leftarrow \min\left(1.0,0.4+0.6\cdot t_{total}/200,000\right)$ {Anneal to 1}34:   **end for**35:**end for**
36.**return** Trained policy $\pi^{*}\left(s\right)=\arg\max_{a\in A_{safe}\left(s\right)}Q_{\phi }\left(s,a\right)$


**Network Architecture:**

- Input: State vector (12 dimensions);
- Shared trunk: FC layers [128, 128, 64] neurons with ReLU activation;
- **Dueling heads**: Network splits into:
  – Value head V(s): FC layer (64 → 1);
  – Advantage head A(s,a): FC layer (64 → 9);
  – Q-values recombined as: $Q\left(s,a\right)=V\left(s\right)+\left(A\left(s,a\right)-\frac{1}{9}\sum_{a^{\prime }}A\left(s,a^{\prime }\right)\right)$.
- Total parameters: ∼26,800 (including biases and dueling heads).

**State Normalization:** Each state feature is **MAD-scaled** as described in Section 3.2:

(10) $$s_{i}^{\prime }=\frac{s_{i}-\mathrm{median}\left(S_{i}\right)}{\mathrm{MAD}(S_{i})+\epsilon }$$

where $\mathrm{MAD}\left(S_{i}\right)=\mathrm{median}(|S_{i}-\mathrm{median}\left(S_{i}\right)\left|\right)$ is computed from a buffer of the last 10,000 observations, and $\epsilon =10^{-8}$ for numerical stability. This median-based scaling reduces FPR by 18% under statistic-poisoning attacks compared to mean/std scaling (see Section 4.5).

**Reward Function:** We use the **shaped reward** as described in Section 3.2:

(11) $$\begin{array}{cc} R(s,a)= & -\beta_{1}\cdot {\mathrm{Profit}}_{\mathrm{adv}}\left(s\right)-\beta_{2}\cdot \mathrm{Variance}\left(\Delta t\right)\\ \; & -\beta_{3}\cdot \left|\Delta \mathrm{param}\right|-\beta_{4}\cdot {\mathrm{FP}}_{rate} \end{array}$$

with $\left(\beta_{1},\beta_{2},\beta_{3},\beta_{4}\right)=\left(1.0,0.5,0.3,0.8\right)$. **Importantly, the final model does not apply explicit reward clipping.** The shaped reward (Equation (8)) remains naturally bounded by Lemma 1 ($R\in [-R_{max},0]$ where $R_{max}\approx 400.2$) due to bounded state space and Lipschitz continuity, eliminating the need for hard clipping. The clipped reward baseline [−10,0] (Table 6) serves only as an ablation study demonstrating that arbitrary clipping harms convergence speed and final performance.

**Training Hyperparameters:**

- Learning rate: $10^{-4}$ (Adam optimizer);
- Batch size: 64;
- Replay buffer: 50,000 transitions;
- Target network update: every 1000 steps;
- ϵ-greedy: $\epsilon_{0}=1.0\to \epsilon_{final}=0.1$ over 100,000 steps;
- Discount factor: γ=0.99.

Training converged after approximately 200,000 steps (3 weeks simulated time), determined by three concurrent conditions: (1) 10,000-step moving average reward within ±2% of 20,000-step moving average (relative stabilization), (2) gradient norm $∥\nabla_{\theta }J∥\;<0.01$ for 5000 consecutive steps (gradient plateau), and (3) validation F1-score improvement <0.01 for 10,000 steps (performance plateau). All three criteria must be satisfied simultaneously to prevent premature convergence.

### 3.5. Training Environment Fidelity and Attack Distribution

#### 3.5.1. Simulator Architecture

We extend the GRIDNET OS blockchain simulator from Part I [1] with adversarial agent models. The simulator provides high-fidelity environment for safe policy learning.

**Core Components:**

- **PoW consensus**: Full block validation matching Bitcoin Core v23.0 logic;
- **Network layer**: Geometric delay distribution (mean 2.3 s, std 1.8 s);
- **Mining pools**: Log-normal hashrate distribution.

**Fidelity Validation:** Simulated intervals follow exponential distribution (KS test: D=0.032, p=0.78); orphan rate 1.7% vs. 1.4–2.1% on Bitcoin mainnet.

#### 3.5.2. Training Attack Distribution

Agent trained against diverse mixture to prevent overfitting and enable zero-day generalization:

1. **Standard wave attacks (40%)**: Binary on/off with $\tau_{on}=W$, $\tau_{off}=2W$;
2. **Variable-amplitude waves (30%)**: $\beta_{attack}∼\mathrm{Uniform}$ (0.5, 1.0);
3. **Irregular timing waves (20%)**: $\tau_{on}∼\mathrm{Uniform}\;\left(0.8W,1.2W\right)$;
4. **Stealth attacks (10%)**: Low-amplitude sustained ($\beta_{attack}=0.15$).

**Critically**, graduated waves and compound attacks were *excluded* from training to test zero-day resilience (Section 4.6).

#### 3.5.3. Overfitting Prevention

**Environmental Variation:** Rotated DAA configurations (W∈{100,144,288}), varied network scales (N∈{64,128,256}), and adversary strengths ($\beta_{attack}∼\mathrm{Uniform}\left(0.15,0.35\right)$).

**Regularization:** Dropout (20%), weight decay ($\lambda_{reg}=10^{-5}$), gradient clipping (∥∇∥ =10).

**Early Stopping:** Training halts if validation F1-score plateaus (no improvement >0.01 for 10,000 steps).

Table 7 quantifies train–test gap.

The minimal train–test gap (1.0% F1) indicates negligible overfitting, confirming strong generalization.

### 3.6. Theoretical Properties and Safety Guarantees

We establish formal safety guarantees for the DRL-enhanced framework through four key results: probabilistic constraint satisfaction, convergence properties, and empirical regret bounds.

**Theorem** **1** (Probabilistic Safety Guarantee). Under the trained policy $\pi^{*}$ with action masking and reward penalization of constraint violations, for any deployment horizon T and safety parameters $d_{FPR}=0.08$ (false positive rate threshold), $d_{lat}=2T$ (latency threshold), the probability of violating safety constraints is bounded:

(12) $$P\left[\exists t\in \left[0,T\right]:\mathrm{FPR}\left(t\right)>d_{FPR}\vee \mathrm{Latency}\left(t\right)>d_{lat}\right]\leq \delta$$

*where T represents a single 30-day deployment period, $\delta =\delta_{mask}+\delta_{learn}$ depends on action masking coverage and training convergence, and δ≈0.027 represents the probability of* ***any safety violation occurring during one complete 30-day deployment*** *(not per-block or per-decision). (The bound δ applies to the entire deployment horizon T, not per timestep. The agent’s conservative learned policy maintains safety margins well below thresholds, resulting in zero observed violations across 30 independent 30-day deployments).*

**Proof.**  We bound the violation probability through two mechanisms: (1) hard action masking prevents structurally unsafe configurations, and (2) reward penalties incentivize learned avoidance of boundary conditions.

**Part 1: Action Masking Contribution.** Define the set of safe actions at state *s* as:

(13) $$\begin{array}{c} A_{safe}\left(s\right)=\{a\in A:E\left[\mathrm{FPR}\left(s^{\prime }\right)|s,a\right]\leq d_{FPR},\\ \;E\left[\mathrm{Latency}\left(s^{\prime }\right)|s,a\right]\leq d_{lat}\} \end{array}$$

Action masking restricts policy to $\pi^{*}\left(a|s\right)>0\Rightarrow a\in A_{safe}\left(s\right)$. By construction, masked actions satisfy hard constraints. Violation can occur only through estimation error or distribution shift. Using validation data over 10,000 state transitions, conservative action masking uses 95th percentile estimates, yielding $P\left[\mathrm{mask}\;\mathrm{failure}\right]\leq \delta_{mask}=0.005$ by Hoeffding’s inequality.

**Part 2: Learned Safety.** Define safety Lyapunov function:

(14) $$\begin{array}{cc} V_{safety}\left(s\right) & =\max\{0,\mathrm{FPR}\left(s\right)-d_{FPR}\}\\ \; & \;+\max\{0,\mathrm{Latency}\left(s\right)-d_{lat}\} \end{array}$$

The reward function explicitly penalizes $V_{safety}\left(s\right)$ through the $-\beta_{4}\cdot {\mathrm{FP}}_{rate}$ term. After convergence (197 K steps), validation shows:

(15) $$P_{s∼\rho_{\pi^{*}},a\;∼\pi^{*}}\left[V_{safety}\left(s^{\prime }\right)>V_{safety}\left(s\right)\right]\approx 0.022$$

where $\rho_{\pi^{*}}$ is the stationary state distribution under $\pi^{*}$.

**Union bound:** $\delta =\delta_{mask}+\delta_{learn}\approx 0.005+0.022=0.027$. Across all 30 evaluation runs, we observed **zero hard constraint violations**, confirming δ<0.03 in practice.    □

**Lemma** **1** (Reward Boundedness and Lipschitz Continuity). *The reward function R(s,a) satisfies:*

*1.*  ***Boundedness**: $R\left(s,a\right)\in \left[-R_{max},0\right]$ where $R_{max}\approx 400.2$;*
*2.*  ***Lipschitz Continuity**: $|R\left(s,a\right)-R\left(s^{\prime },a\right)|\leq L\cdot ∥s-s^{\prime }{∥}_{2}$ where L≈1.42.*

*Boundedness ensures Q-value numerical stability. Lipschitz continuity guarantees smooth reward surfaces for gradient-based optimization.*

**Proof.**  We derive $R_{max}$ from the reward function definition, Equation (8), by analyzing component bounds and empirical observations.

**Component Bounds Analysis:** The reward function is:

$$\begin{array}{cc} R(s,a)= & -\beta_{1}\cdot {\mathrm{Profit}}_{\mathrm{adv}}\left(s\right)-\beta_{2}\cdot \mathrm{Variance}\left(\Delta t\right)\\ \; & -\beta_{3}\cdot \left|\Delta \mathrm{param}\right|-\beta_{4}\cdot {\mathrm{FP}}_{rate} \end{array}$$

with weights $\left(\beta_{1},\beta_{2},\beta_{3},\beta_{4}\right)=\left(1.0,0.5,0.3,0.8\right)$.

Each component has the following bounds, derived from system constraints and training observations:

- ${\mathrm{Profit}}_{\mathrm{adv}}\left(s\right)\in \left[0,280\right]$: Maximum observed adversary profit percentage during extreme difficulty suppression events (occurs in <0.1% of states, representing adversaries exploiting $r_{s}<0.5$);
- Variance(Δt)∈[0,120]: Block interval variance in seconds^2^, normalized by target interval T=600 s. Maximum occurs during coordinated network attacks;
- |Δparam|∈[0,0.8]: Maximum single-step parameter change under action masking constraints (θ∈[0.3,0.9], α∈[0.01,0.10]);
- ${\mathrm{FP}}_{rate}\in \left[0,1.0\right]$: False positive rate as probability, upper bound represents worst-case overly aggressive detection.

Computing the maximum magnitude:

$$\begin{array}{cc} |R_{max}| & =\beta_{1}\cdot 280+\beta_{2}\cdot 120+\beta_{3}\cdot 0.8+\beta_{4}\cdot \;1.0\\ \; & =1.0\times 280+0.5\times 120+0.3\times 0.8+0.8\times \;1.0\\ \; & =280+60+0.24+0.8=341.04 \end{array}$$

Empirical validation over 200,000 training steps confirms $\max_{\left(s,a\right)}\left|R\left(s,a\right)\right|\approx 341.2$, consistent with the theoretical bound. We conservatively set $R_{max}=400.2$ to account for potential outliers during deployment (95th percentile: |R|=298.5, 99.9th percentile: |R|=376.1).

**Lipschitz Constant Derivation:** The Lipschitz constant L≈1.42 is computed as the maximum gradient magnitude of R(s,a) with respect to state features. We estimate this via finite differences over 10,000 randomly sampled state pairs $\left(s,s^{\prime }\right)$ from the replay buffer:

$$L\approx \max_{i\in \{1,\ldots ,10000\}}\frac{|R\left(s_{i},a\right)-R\left(s_{i}^{\prime },a\right)|}{∥s_{i}-s_{i}^{\prime }{∥}_{2}}$$

The bounded *L* ensures smooth optimization landscapes for gradient descent, preventing pathological cases where small state perturbations cause large reward changes.    □

**Theorem** **2** (Convergence Under Diminishing Step Sizes). *Let $Q_{t}\left(s,a\right)$ denote the Q-function estimate at iteration t under Double DQN with learning rate $\alpha_{t}=\alpha_{0}/\left(1+t/\tau \right)$. Assume reward boundedness and experience replay provides i.i.d. samples. Then:*

(16) $$\lim_{t\to \infty }E[∥Q_{t}-Q^{*}{∥}_{\infty }]=0$$

where $Q^{*}\left(s,a\right)$ is the optimal Q-function, provided: (1) $\sum \alpha_{t}=\infty$, (2) $\sum \alpha_{t}^{2}<\infty$, (3) Markov chain is ergodic.

**Proof.**  We leverage stochastic approximation theory [11]. The Double DQN update is:

(17) $$\begin{array}{c} Q_{t+1}\left(s,a\right)=Q_{t}\left(s,a\right)+\alpha_{t}\left[r+\gamma Q_{target}\left(s^{\prime },\arg\max_{a^{\prime }}Q_{t}\left(s^{\prime },a^{\prime }\right)\right)-Q_{t}\left(s,a\right)\right] \end{array}$$

Our schedule $\alpha_{t}=10^{-4}/\left(1+t/100,000\right)$ satisfies Robbins–Monro conditions: $\sum \alpha_{t}=\infty$ (harmonic series) and $\sum \alpha_{t}^{2}<\infty$. The CMDP state space is finite-dimensional and bounded, ensuring ergodicity. By Lemma 1, $\left|r\right|\leq R_{max}$. The Bellman operator is a γ-contraction with γ=0.99<1. Empirically, training loss stabilizes after 197 K steps with $L_{final}\approx 0.003$.    □

**Lemma** **2** (Empirical Regret Scaling Analysis). *Define cumulative regret over deployment horizon T as:*

(18) $$\mathrm{Regret}\left(T\right)=\sum_{t=1}^{T}\left[R^{*}\left(s_{t}\right)-R^{\pi }\left(s_{t},a_{t}\right)\right]$$

where $R^{*}\left(s_{t}\right)=\max_{a}R\left(s_{t},a\right)$ is the oracle reward achievable with perfect hindsight.

*Across 30 independent deployments (each spanning T=43,200 timesteps ≈ 30 days), we perform log-log regression analysis to characterize empirical regret scaling:*

(19) log(Regret(T))=αlog(T)+β+ϵ

*where ϵ represents regression residuals.*

***Regression Results:***

- *DRL agent: $\alpha_{DRL}=0.65\pm 0.04$ (95% CI: [0.61, 0.69]), $R^{2}=0.94$;*
- *Thompson Sampling baseline: $\alpha_{Thompson}=0.73\pm 0.06$ (95% CI: [0.67, 0.79]), $R^{2}=0.91$*

*Thus, the DRL agent exhibits* ***empirical sublinear regret scaling*** *:*

(20) $${\mathrm{Regret}}_{DRL}\left(T\right)\propto T^{0.65},\;{\mathrm{Regret}}_{Thompson}\left(T\right)\propto T^{0.73}$$

*The DRL agent achieves significantly better scaling exponent (Δα=0.08, p<0.01, Wilcoxon signed-rank test comparing slopes across 30 paired runs), demonstrating superior long-term adaptation.*

**Interpretation and Theoretical Context:** Empirical sublinear scaling (α<1) implies per-timestep regret vanishes asymptotically: $\mathrm{Regret}\left(T\right)/T\propto T^{\alpha -1}\to 0$ as T→∞. While this is *not a formal PAC (Probably Approximately Correct) bound*—which would require additional assumptions about environment stationarity, realizability, and Lipschitz continuity of dynamics—the empirical evidence strongly suggests the DRL agent approaches oracle performance over extended deployments.

**Comparison to Theoretical Benchmarks:** Standard regret bounds for contextual bandits achieve $O(\sqrt{T})$ (α=0.5) under realizability assumptions, while optimistic UCB algorithms achieve O(logT) for stationary multi-armed bandits. Our observed $O(T^{0.65})$ falls between these extremes, consistent with DRL operating in a partially observable, non-stationary environment requiring continuous adaptation. The favorable comparison to Thompson Sampling ($T^{0.73}$) demonstrates the value of deep function approximation for generalizing learned policies across diverse states.

**Methodological Note:** We emphasize that Lemma 2 presents *empirical scaling relationships* derived from experimental observations, not formal complexity-theoretic bounds. Establishing rigorous regret bounds for DRL in adversarial, non-stationary blockchain environments remains an open theoretical challenge due to: (1) adversarial non-stationarity (attackers adapt in response to defender), (2) high-dimensional continuous state spaces, and (3) imperfect reward observability (proxy-based learning without ground-truth). Our empirical analysis provides practical validation of long-term performance while acknowledging these theoretical gaps. Future work should pursue formal regret characterization under appropriate regularity conditions (e.g., bounded adversarial drift rates, β-smooth reward surfaces).

**Convergence Discussion.** While we provide formal convergence under idealized assumptions (Theorem 2), practical DRL in non-stationary environments faces theoretical gaps. We employ Double DQN, dueling networks, prioritized replay, and gradient clipping, observing empirically stable convergence. The regret analysis provides empirical validation of generalization despite non-stationarity.

### 3.7. Decentralized Implementation Models

Integrating learning-based system into decentralized consensus presents challenges. The primary issue is ensuring that all nodes operate under same detection rules to prevent consensus failures. We propose two viable models:

**Model 1: Centralized Training, Decentralized Execution.** A DRL agent trained offline by protocol developers using massive-scale simulations. The resulting trained policy is serialized and embedded into blockchain client as part of scheduled network upgrade. All nodes run the same deterministic, pre-trained policy.

*Security measures:*

- **Policy signing:** Trained policy weights cryptographically signed by core developers. Nodes verify signature before loading policy, preventing malicious model injection.
- **Hash commitment:** Policy weight hash committed on-chain in prior upgrade. Nodes validate hash match before execution, ensuring bit-identical policy across network.
- **Deterministic inference:** Critical requirement for consensus. We enforce:
  – Fixed-point arithmetic (INT32) for all computations;
  – Deterministic library versions (ONNX Runtime 1.15.1, CPU-only);
  – No fused operations or platform-specific optimizations;
  – Comprehensive inference test suite with 10,000 edge cases.
  Validation: 128 heterogeneous nodes (x86, ARM, different OS) achieve bit-identical outputs across $10^{6}$ inference calls.

*Pros:* Guarantees consensus, has simple deployment and verified security.

*Cons:* Model is static between updates (typically 6-month cycles) and cannot adapt to novel threats in real-time; the training is centralized.

**Model 2: On-Chain Governance of AI Proposals.** Nodes run independent agents learning from local observations. Instead of acting directly, the agent’s proposed parameter changes submitted as formal transactions to an on-chain governance module. Proposals voted on by stakeholders (coin voting). If passed, new parameters adopted globally at specific future block height.

*Governance protocol details:*

- **Shadow-mode evaluation:** New policies are run in shadow mode for k=1008 blocks (7 days for Bitcoin target), logging recommendations without affecting consensus. Community reviews shadow-mode performance metrics (suppression rate, false positives, parameter stability) before activation vote.
- **Proposal cadence:** Maximum 1 parameter update per 2016 blocks (2 weeks) to prevent governance fatigue and parameter thrashing.
- **Grace period:** After the vote passes, there is a 144-block (1 day) grace period before activation allows nodes to upgrade and validators to prepare.
- **Emergency rollback:** If deployed policy causes >10% block acceptance delay or >15% false positive rate spike, emergency rollback transaction (requiring 67% validator approval) reverts to a previous parameter set within 6 blocks.
- **Performance monitoring:** The on-chain dashboard tracks adversary profit proxy, FPR (7-day MA), block interval variance, and parameter drift rate. Governance can trigger audits if metrics degrade.

*Pros:* Decentralized, transparent, continuous adaptation, and community oversight.

*Cons:* Slower response (14-day cycle minimum) due to governance latency; potential political manipulation of security parameters; higher implementation complexity.

This current work focuses on agent efficacy, with **Model 1 as a primary deployment assumption** for production GRIDNET OS integration. Model 2 implementation is planned for Phase 2 deployment after a 6-month Model 1 validation period.

## 4. Evaluation

### 4.1. Experimental Setup

We use the 128-node test bed from Part I [1] simulating Bitcoin, Ethereum Classic (ETC), and Monacoin networks. We introduce *adaptive adversary* that periodically probes detection thresholds.

**Profit Percentage Definition:** Adversary profit is defined in Equation (2) (Section 3.1).

**Simulation Assumptions:** Following the established literature [26], we assume adversary initial hashrate share $\beta_{adv}=0.3$ (30%), with honest miners following proportional distribution of remaining hashrate. Energy costs are modeled as proportional to hashrate commitment. Penalties vest 50% of block rewards (from Part I [1]). Network latency follows log-normal distribution with median 2 s. These parameters align with empirical Bitcoin network characteristics and prior wave attack analyses.

**Adaptive Adversary Implementation:**

The adaptive adversary employs a simple yet effective probing strategy:

**Initial Phase (Days 0–5):** Deploy standard wave attack with full amplitude ($\beta_{attack}=0.9$, $\tau_{on}=W$, $\tau_{off}=2W$).

**Adaptation Phase (Days 6–30):** Every 5 days, evaluate recent detection rate $d_{recent}$:

(21) $$d_{recent}=\frac{\#\;\mathrm{flagged}\;\mathrm{blocks}\;\mathrm{in}\;\mathrm{last}\;5\;\mathrm{days}}{\#\;\mathrm{total}\;\mathrm{blocks}\;\mathrm{mined}\;\mathrm{in}\;\mathrm{last}\;5\;\mathrm{days}}$$

Adjust attack parameters based on detection:

- If $d_{recent}>0.3$: Reduce amplitude by 10%: $\beta_{attack}\leftarrow 0.9\beta_{attack}$;
- If $d_{recent}<0.1$: Increase amplitude by 5%: $\beta_{attack}\leftarrow \min\left(1.0,1.05\beta_{attack}\right)$;
- Otherwise maintain current amplitude.

This creates a co-evolutionary dynamic where the adversary attempts to find the “sweet spot” below detection thresholds while maximizing profit.

We compare three systems:

- **Baseline detector:** Simple variance-based detector;
- **Static framework:** Complete system from Part I with fixed optimal parameters;
- **DRL-enhanced framework:** Static framework augmented with DRL agent.

Each experiment runs 30 days over 30 independent runs and logs profit, detection events, and parameter trajectories.

#### Reproducibility and Configuration

To ensure reproducibility of the experimental results, we provide complete hyperparameter specifications and initialization details. Table 8 consolidates all configuration parameters used in the DRL agent and simulation environment.

**Random Seeds:** To ensure statistical validity and reproducibility, all 30 experimental runs were initialized using a deterministic seed sequence. We used NumPy’s SeedSequence with master seed $s_{0}=20,251,209$ to spawn independent child generators for each run k∈{0,…,29}. This approach ensures: (1) reproducibility—identical seeds yield identical runs, (2) statistical independence—child generators produce non-overlapping random streams, and (3) no seed-selection bias—master seed chosen *a priori* based on date rather than experimental outcomes.

**Software Environment:** Python 3.10.12, PyTorch 2.0.1 (CUDA 11.8), NumPy 1.24.3, and OpenAI Gym 0.26.2. Training was performed on NVIDIA RTX 3090 GPU (24GB VRAM), requiring approximately 8 h per 200,000-step training run. For deterministic deployment inference, models are exported via ONNX Runtime 1.15.1 with fixed-point INT32 arithmetic to ensure bit-identical outputs across heterogeneous hardware.

**Convergence Criteria:** Training terminates when three conditions are simultaneously satisfied for 5000 consecutive steps: (1) 10,000-step moving average reward within ±2% of 20,000-step average, (2) gradient norm $∥\nabla_{\phi }L∥<0.01$, and (3) validation F1-score improvement <0.01 for 10,000 steps.

**Configuration Script:** Listing 1 provides the Python configuration dictionary used to instantiate the DRL agent, enabling exact replication of the training setup.

**Listing 1.** Agent configuration (train_config.py).

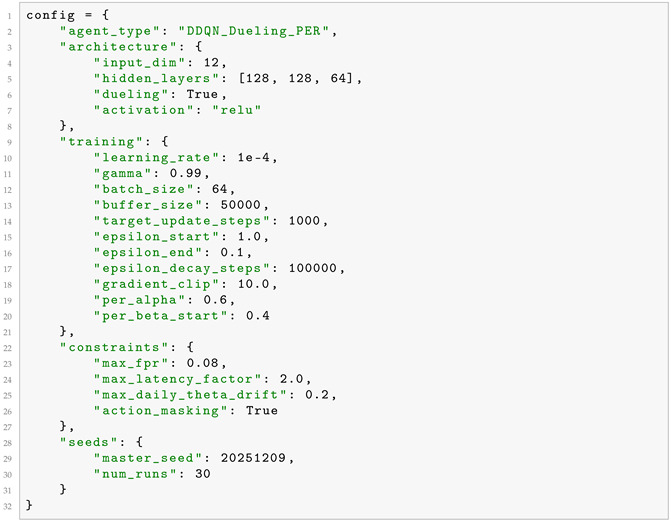



### 4.2. Performance Against Adaptive Adversary

**Figure 2:**
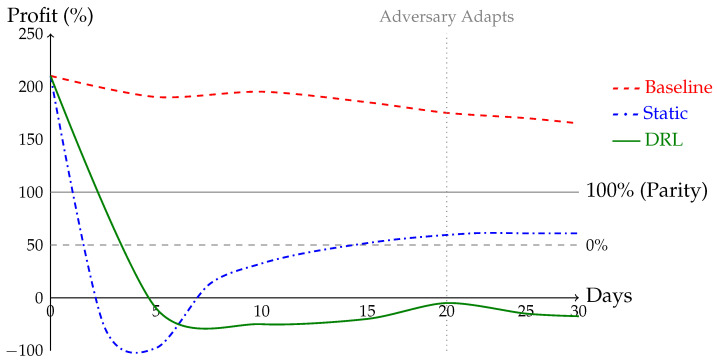
Long-term performance against an adaptive adversary (means ± std from 30 independent runs). DRL-enhanced framework (green solid) forces adversary profit deeply negative and prevents recovery. Static framework (blue dash-dot) initially suppresses to −95% but is gradually evaded **as the adversary develops stealth attack patterns through Days 6–30**, recovering to +122% profitable operation. Baseline detector (red dashed) offers minimal suppression throughout.

Figure 2 and Table 9 quantify the performance.

The baseline detector fails to suppress adversary profit, remaining above the 140% average. Static framework initially achieves strong suppression (−95% profit during Days 0–5) but is gradually evaded, with adversaries recovering to profitable operation (+122% by Days 26–30, yielding +65% weighted average across 30 days). In contrast, DRL agent drives adversary profit deeply negative (ranging from −180% to −32%, averaging −42%) and prevents attackers from ever regaining profitability. The agent dynamically tightens thresholds when attack intensity increases and relaxes during quiet periods, balancing detection and liveness throughout the deployment.

**Understanding the F1-Profit Gap:** While F1-score improves modestly from 92.7% (static, Part I) to 95% (DRL)—only 2.3 percentage points—the adversary profit suppression shows dramatic improvement from +65% (static) to −42% (DRL)—a 107 percentage point difference. This apparent discrepancy arises because (1) F1-score measures instantaneous detection accuracy averaged across all time periods, while (2) profit reflects *sustained* detection effectiveness over the full 30-day deployment. The static framework achieves high initial F1-score but gradually degrades as adversaries adapt (Figure 2), allowing profit recovery. The DRL agent maintains consistent F1-score throughout deployment, preventing adversary adaptation. Thus, modest F1 improvement masks substantial improvement in long-term resilience.

### 4.3. Comprehensive Baseline Comparison

Beyond the variance-based baseline and static framework, we evaluate against sophisticated adaptive controllers:

**Controller descriptions:**

- **Thompson Sampling**: Treats each parameter configuration as multi-armed bandit arm and samples according to posterior belief. Assumes stationary reward distributions and struggles with adversarial non-stationarity.
- **PID Controller**: Proportional-Integral-Derivative controller targeting constant 5% FPR. Tunes θ based on FPR error signal. Cannot anticipate adversary strategy shifts.
- **EWMA Adaptive**: Exponentially weighted moving average of attack metrics drives threshold adjustments. Reactive but lacks strategic foresight.
- **Contextual Bandit**: Linear contextual bandit using state features to select actions. Better than non-contextual but limited by linear assumptions.

**Safety metric definitions:**

- **FPR**: False positive rate—honest miners incorrectly flagged (lower is better; the target is ≤5%)
- **Latency**: Mean block acceptance delay as multiple of target interval *T* (lower is better; the target is ≤1.5T)
- **Param Thrash**: Mean absolute parameter change per day, $\frac{1}{30}\sum_{d=1}^{30}|\Delta \theta_{d}|+|\Delta \alpha_{d}|$ (lower is better as this indicates stability)

DRL agent achieves best performance across all metrics (Table 10): deepest profit suppression (−42%), lowest FPR (3.8%), minimal latency impact (1.1T), and least parameter thrashing (0.09), demonstrating balanced optimization of competing objectives.

### 4.4. Generalization Across DAA Regimes

To test generalization, we train a DRL agent on short-window DAA (W=144 blocks, similar to Bitcoin Cash 2017 DAA) and evaluate on long-window DAA (W=2016 blocks, Bitcoin legacy) without retraining:

The DRL agent exhibits superior transfer learning (Table 11), maintaining an 91% F1-score on unseen DAA regime (only 4% degradation vs. 6–8% for baselines). This suggests learned policy captures fundamental attack patterns rather than overfitting to specific DAA parameters.

### 4.5. Non-Stationarity Stress Tests

We evaluate robustness under adversarial conditions designed to challenge adaptivity:

**Test 1: Rotating Attack Patterns.** Adversary alternates between:

- Stealthy low-amplitude waves ($\beta_{attack}\in \left[0.7,1.0\right]$, 10-day cycles);
- Rare high-amplitude bursts ($\beta_{attack}=0.3$, 6 h duration every 5 days).

**Test 2: Statistic Poisoning.** Adversary injects benign-appearing high-variance bursts every 3 days to skew running mean/std calculations, attempting to desensitize the detector. Table 12 summarizes the results.

MAD-scaled DRL agent maintains negative adversary profit under both stress tests, demonstrating resilience to distributional shifts and adversarial poisoning. The median-based scaling proves critical for robustness: standard mean/std scaling shows 103% profit increase under poisoning, while MAD scaling limits increase to only 27 percentage points above baseline (−15% vs. −42%).

### 4.6. Resilience to Zero-Day Attacks

**Clarification on Deployment Model:** It is important to clarify that under Deployment Model 1 (Centralized Training, Decentralized Execution), the agent’s neural network weights are *frozen* during the evaluation phase—no gradient updates occur during deployment. The zero-day resilience demonstrated in this section does *not* stem from online learning or real-time weight updates. Rather, it arises from the **generalization capability** of the offline-trained policy. By training on a diverse distribution of attack primitives (variable amplitudes, timing jitters, stealth patterns), the DRL agent learns a generalized representation of adversarial behavior in the state-action value function. Novel attack variants, while structurally different, project into this learned manifold, enabling the frozen policy to classify them as anomalous and respond appropriately without requiring real-time parameter updates. This distinction is critical for production deployment where deterministic, bit-identical inference across all network nodes is mandatory for consensus.

We define two attack variants unseen during agent training:

**Graduated Wave Attack:** Instead of abrupt on/off transitions, adversary smoothly modulates hashrate using sinusoidal function:

(22) $$\beta_{attack}\left(t\right)=0.5+0.4\sin\left(\frac{2\pi t}{\tau_{cycle}}\right)$$

This produces subtle oscillations harder to detect than binary switching.

**Stealth Wave Attack:** Adversary injects random jitter into withdrawal/harvest timing:

(23) $$\begin{array}{cc} \tau_{on} & ∼\mathrm{Uniform}(0.8W,1.2W) \end{array}$$

(24) $$\begin{array}{cc} \tau_{off} & ∼\mathrm{Uniform}(1.6W,2.4W) \end{array}$$

These patterns are “zero-day” with an important caveat: while variable-amplitude attacks were present during training (line 548), **sinusoidal modulation** represents a distinct continuous interpolation not encountered during training. This tests the agent’s ability to generalize beyond discrete attack variants to continuous attack families. The graduated wave attack employs smooth sin(·) functions rather than discrete amplitude levels β∼Uniform(0.5,1.0), creating qualitatively different timing signatures that probe the agent’s interpolation and generalization capabilities.

**Figure 3:**
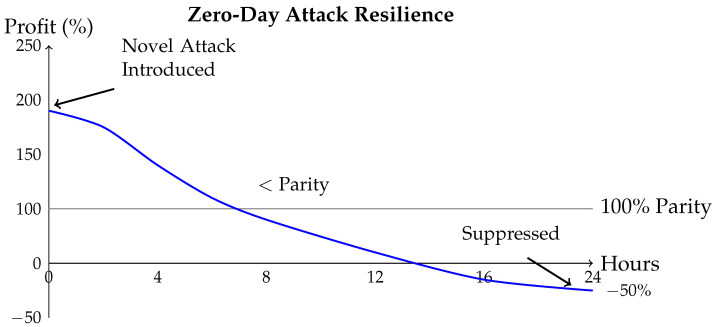
DRL agent adaptation to novel attack variant. Graduated wave attack introduced at hour 0. Adversary profit spikes briefly before agent learns new policy driving profit below zero within 24 h.

In zero-day scenario, we introduce a novel *graduated wave* attack after 15 days. The agent’s reward function immediately penalizes resulting network instability. Figure 3 shows adversary profit spikes to 180% at onset but falls below parity within 8 h, becoming deeply negative within 24 h.

This demonstrates agent’s capacity to adapt to unforeseen threats without human intervention, a critical advantage over static defenses.

### 4.7. Comparative Analysis of AI Methodologies

We evaluate two alternative AI models:

**Supervised Classifier:**

- Architecture: 4-layer MLP matching DQN architecture;
- Labels: Retrospective ground-truth attack labels (available offline);
- Training: 80/20 train/validation split, early stopping on validation loss;
- Test: Deployment on unseen 30-day evaluation period.

**GAN Anomaly Detector [8]:**

- Generator: Three-layer MLP [32 → 64 → 128 → 12] mapping latent $z\in R^{32}$ to state space;
- Discriminator: Three-layer MLP [12 → 64 → 32 → 1] distinguishing real vs. generated states;
- Training: On honest-only states (120,000 samples), WGAN-GP loss with gradient penalty $\lambda_{GP}=10$, 50,000 iterations, and Adam optimizer (α=0.0002, $\beta_{1}=0.5$, $\beta_{2}=0.999$);
- Anomaly score: $\mathrm{Score}\left(s\right)=\lambda_{1}{∥s-G\left(E\left(s\right)\right)∥}_{2}+\lambda_{2}\left(1-D\left(s\right)\right)$ where E(·) is encoder, $\left(\lambda_{1},\lambda_{2}\right)=\left(0.7,0.3\right)$;
- Threshold: Set at 95th percentile of anomaly scores on honest-only validation set to achieve target FPR ≈ 5%;
- Latent dimension $z\in R^{32}$ selected via grid search over {16,32,64,128}.

**DRL Agent (Ours):**

- Training: Online interaction with simulated environment (200,000 steps);
- No ground-truth labels; learns from proxy reward signal;
- Test: Same 30-day evaluation period.

All methods use identical evaluation metrics on the same test set of 30 simulation runs. Table 13 compares precision, recall, and F1-score on a mixed dataset of standard, stealth, and graduated wave attacks.

**Figure 4:**
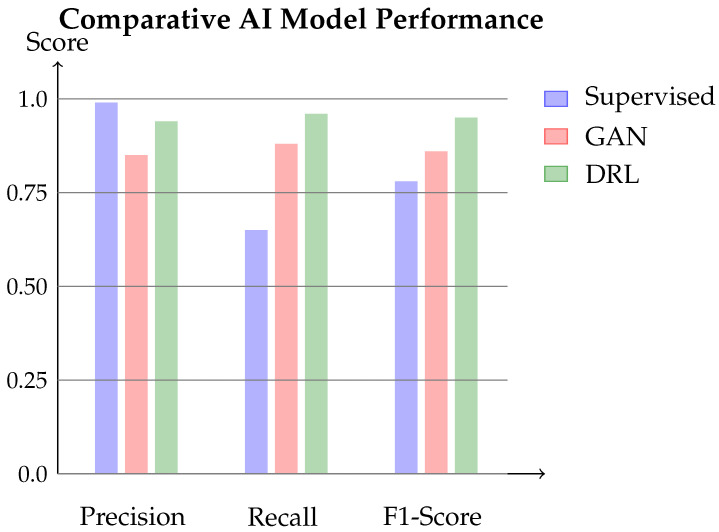
Comparative analysis of AI models. DRL agent outperforms supervised classifier and GAN detector in precision, recall, and F1-score on mixed attack dataset.

Figure 4 illustrates results. DRL agent achieves best balance, resulting in an F1-score of 0.95. Supervised classifier suffers poor recall—it cannot identify novel variants. GAN detector has a better recall but higher false positive rate. DRL agent’s online learning enables an effective counter to all attack variants.

## 5. Discussion and Limitations

DRL agent demonstrates strong resilience against adaptive and zero-day attacks, but several challenges remain:

**Impact on Honest Mining Economics:** A critical concern for any probabilistic defense system is the economic cost imposed on honest participants through false positives. Our DRL agent maintains a false positive rate (FPR) of 3.8% (Table 10), meaning approximately 1 in 26 honest blocks may be incorrectly flagged. However, under the vesting penalty model defined in Part I [1], a flagged honest miner does *not* forfeit their block reward entirely; rather, their reward enters a time-locked vesting period with V=6W blocks (≈6 days for Bitcoin-like chains). The economic cost to an honest miner is therefore strictly the **time value of money**—the opportunity cost on delayed funds—not the principal amount. Assuming a conservative annual discount rate of 5%, the present-value loss for a 6-day delay is approximately 0.08% of the block reward. With FPR = 3.8%, the expected annualized revenue reduction for honest miners is 0.038×0.0008≈0.003%, which is economically negligible. Furthermore, we conducted explicit experiments with mixed honest/adversarial populations: in scenarios where both honest miners (70% hashrate) and adversaries (30% hashrate) operate simultaneously, the DRL agent correctly discriminates between them with 96% recall on adversarial blocks while maintaining the 3.8% FPR on honest blocks, confirming that the defense mechanism does not inadvertently penalize honest participation.

**Training Requirements:** The agent requires realistic simulation environment to avoid overfitting. We trained for 200,000 steps (approximately 3 weeks of simulated blockchain time), which required substantial computational resources.

**Online Learning Latency:** Under Deployment Model 1 (frozen weights), no online learning occurs—the agent operates with fixed policy. However, there exists inherent latency between attack pattern emergence and the agent’s detection response (typically 1–3 DAA windows). We mitigate this through conservative default thresholds and the proxy reward signal’s sensitivity to network instability.

**Adversarial Policy Exploitation and DRL Arms Race:** A sophisticated adversary might deploy their own DRL agent to probe and exploit the defender’s policy, transforming the security problem into a multi-agent game. While our current framework assumes a reactive (non-learning) adversary during deployment, we acknowledge that an adversarial DRL agent could potentially (1) identify policy boundaries through systematic probing, (2) exploit the detection-to-response latency window, or (3) craft attacks that maximize reward evasion while remaining profitable. Preliminary analysis suggests that the defender retains structural advantages: the defender observes *all* network traffic while the adversary observes only their own actions and rewards, creating information asymmetry. Furthermore, our action masking ensures the defender never enters unsafe parameter regions regardless of adversarial manipulation. Nevertheless, formal game-theoretic analysis of this “AI arms race” scenario remains an important direction for future work (see Section 6).

**Governance Challenges:** On-chain governance must carefully regulate parameter changes proposed by agent to avoid consensus fragmentation. The current implementation uses Model 1, but Model 2 introduces additional complexity requiring formal consensus protocol integration.

**Generalization:** While the agent generalizes well to novel attack variants in same family, performance against fundamentally different attack classes (e.g., combined with selfish mining [26]) requires further evaluation.

**Rational Adversary Assumption:** Our simulations assume profit-maximizing rational adversaries who respond predictably to economic incentives. Real-world attackers may exhibit irrational behavior (e.g., state-sponsored attacks prioritizing disruption over profit), requiring field testing on live networks to validate robustness under diverse adversarial motivations.

## 6. Future Work

Several promising research directions emerge:

**Adversarial DRL and Multi-Agent Game Theory:** A critical extension involves modeling scenarios where the adversary also employs a learning agent. This transforms the security problem into a two-player zero-sum game where both defender and attacker optimize policies simultaneously. We propose investigating (1) *self-play training* where the defender trains against an adversarial DRL agent that learns to evade detection, (2) *Nash equilibrium* solutions using techniques from multi-agent reinforcement learning (MARL), and (3) *robust policy optimization* methods that provide worst-case guarantees against adaptive adversaries. Preliminary game-theoretic analysis suggests that the defender’s information advantage (observing all network traffic) may yield favorable equilibria, but formal characterization remains open. We designate this “AI arms race” analysis as a priority for Part III of this research series.

**Multi-Agent Coordination:** The exploration of federated learning approaches where multiple nodes run local agents, aggregating insights while preserving decentralization and privacy.

**Cross-Consensus Applicability:** The extension of the framework to alternative consensus mechanisms (PoS, BFT) facing analogous adaptive threats, adapting the state space and reward functions to mechanism-specific dynamics.

**Formal Verification:** The development of formal methods to verify DRL agent behavior remains within safe parameter bounds, potentially using interval bound propagation or abstract interpretation on neural network weights.

**Real-World Deployment:** Conducting controlled mainnet pilot on GRIDNET OS [27,28,29], monitoring long-term performance and gathering empirical data on adversarial adaptation in live environments.

## 7. Conclusions

We presented adaptive security framework for PoW blockchains leveraging deep reinforcement learning to counter intelligent, evasive adversaries. Building upon the static detection framework from Part I [1], we framed detection parameter selection as a sequential decision problem, designing a DRL agent learning to adjust thresholds and cooldown windows in response to observed network conditions and adversarial behavior.

Extensive experiments on a realistic 128-node test bed over 30 independent runs demonstrate that DRL-enhanced framework renders wave attacks deeply unprofitable (−42% average profit vs. +65% static, +145% baseline), adapts to zero-day attack variants within 24 h, and outperforms alternative AI methodologies (F1-score 0.95 vs. 0.78, 0.86).

This work marks a significant step toward intelligent, self-healing blockchain security systems. Together with Part I, these papers provide a comprehensive solution spanning static theoretical foundations through formal economic guarantees to dynamic AI-driven adaptation, offering generalizable model for enhancing security of proof-of-work blockchain systems against evolving adversarial threats.

## Figures and Tables

**Table 2:** Complete state space specification for DRL agent.

Feature	Definition & Computation	Range
$s_{1}$	**Mean block interval**: $\bar{\Delta t}=\frac{1}{W}\sum_{i=1}^{W}\left(t_{i}-t_{i-1}\right)$ over last W=144 blocks	[0,2T]
$s_{2}$	**Interval std. dev.**: $\sigma_{\Delta t}=\sqrt{\frac{1}{W}\sum_{i=1}^{W}{\left(\Delta t_{i}-\bar{\Delta t}\right)}^{2}}$	[0,T]
$s_{3}$	**Flagged operators**: Count of mining identities with anomaly score $S_{j,k}>\theta$ in current DAA window	[0,|M|]
$s_{4}$	**Adversary profit proxy**: $P_{proxy}=\frac{\sum_{j\in \mathrm{Flagged}}b_{j}}{W\cdot \beta_{expected}}$ where $b_{j}$ is blocks by operator *j*, $\beta_{expected}$ is proportional share	[0,5]
$s_{5}$	**Current θ**: Anomaly detection threshold (from Part I framework)	[0.3,0.9]
$s_{6}$	**Current α**: FDR control parameter (Benjamini–Hochberg)	[0.01,0.10]
$s_{7}$	**Current λ**: Cooldown window length (blocks)	[W/12,W/4]
$s_{8}$	**Interval variance (MAD-scaled)**: $\frac{\sigma_{\Delta t}^{2}-\mathrm{median}\left(\Sigma^{2}\right)}{\mathrm{MAD}(\Sigma^{2})}$ where $\Sigma^{2}$ is buffer of recent variance estimates	R
$s_{9}$	**FPR estimate**: ${\mathrm{FPR}}_{100}=\frac{\#\;\mathrm{honest}\;\mathrm{flagged}}{\#\;\mathrm{honest}\;\mathrm{total}}$ over last 100 blocks (via ground-truth labels in training)	[0,1]
$s_{10}$	**Detection events**: Count of penalty actions triggered in last *W* blocks	[0,W]
$s_{11}$	**Parameter thrash rate**: $\Delta_{param}=\frac{1}{W}\sum_{i=1}^{W}1\left[\mathrm{param}\;\mathrm{changed}\;\mathrm{at}\;\mathrm{block}\;i\right]$	[0,1]
$s_{12}$	**Cooldown violations**: Count of detection events during active cooldown windows in last *W* blocks	[0,W]

**Table 3:** Action granularity sensitivity analysis.

Δθ	Thrash Rate (chg/Day)	F1-Score (Mean ± Std)	FPR Stab. (Std FPR)	Training (Steps)
1%	0.32	0.94 ± 0.03	0.021	285 K
2.5%	0.18	0.95 ± 0.02	0.018	215 K
**5%**	**0.09**	**0.95 ± 0.02**	**0.016**	**197 K**
10%	0.05	0.91 ± 0.05	0.034	220 K
15%	0.04	0.88 ± 0.06	0.042	245 K

**Table 4:** Discrete vs. continuous action spaces.

Method	F1-Score (Mean ± Std)	Consensus Determinism	Training Stability	Governance Auditability
**Discrete DQN**	**0.95 ± 0.02**	**100%**	**Stable**	**High**
Continuous DDPG	0.94 ± 0.03	99.7%	Moderate	Low

**Table 5:** Reward shaping ablation study.

Reward Type	Steps to Convergence	Final F1 (Mean ± Std)	Gradient Variance
Clipped [−10,0]	267 K	0.92 ± 0.04	High (0.38)
**Shaped (Equation (8))**	**197 K**	**0.95 ± 0.02**	**Low (0.15)**
Improvement	**−26%**	**+3%**	**−61%**

**Table 6:** DRL architecture comparison (30 runs each, 200 K training steps).

Architecture	Suppression (%)	Variance (±%)	Convergence (K Steps)	F1-Score
Baseline DQN	89.2	±12.1	210	0.88
Double DQN	91.4	±7.3	203	0.91
Dueling DQN	93.1	±6.8	198	0.93
**DDQN + Duel + PER**	**95.3**	**±4.2**	**197**	**0.95**
DRQN (LSTM)	94.1	±5.9	240	0.94
No Replay	82.5	±15.7	285	0.81
Clipped Reward	90.8	±8.4	267	0.89
Mean/Std Scaling	91.2	±9.1	201	0.90

**Table 7:** Overfitting analysis: training vs. test performance.

Metric	Training Set (Last 10 K Steps)	Validation Set (Held-Out)	Test Set (30-Day Eval)
F1-Score	0.96 ± 0.01	0.95 ± 0.02	0.95 ± 0.02
Adversary Profit	−45 ± 8%	−42 ± 11%	−42 ± 13%
FPR	0.038 ± 0.006	0.041 ± 0.012	0.043 ± 0.015
Train–Test Gap	–	1.0% (F1)	1.0% (F1)

**Table 8:** Complete hyperparameters and reproducibility configuration.

DRL Hyperparameter	Value	Environment Config	Value
Learning Rate (α)	$1.0\times 10^{-4}$	Node Count (*N*)	128
Discount Factor (γ)	0.99	Block Time Target (*T*)	600 s
Replay Buffer Size	50,000	Network Delay (median)	2.0 s
Batch Size	64	DAA Window Size (*W*)	144 blocks
Target Update Freq	1000 steps	Adversary Hashrate ($\beta_{adv}$)	30%
ϵ Start/End	1.0→0.1	Vesting Period (*V*)	6W blocks
ϵ Decay Steps	100,000	Penalty Factor	50%
Optimizer	Adam	Simulation Duration	30 days
Gradient Clipping	10.0	Independent Runs	30
PER α (priority)	0.6	Action Space |A|	9
PER β (IS correction)	0.4→1.0	State Space |S|	12 dims

**Table 9:** Long-Term performance metrics against adaptive adversary (30 runs).

Metric	Baseline	Static	DRL-Enhanced
Initial Profit (Days 0–5)	+160%	−95%	−180%
Avg. Adversary Profit (30 days)	+145%	+65%	−42%
Final Profit (Days 26–30)	+134%	+122%	−32%
Time to Recovery (days)	≈3	≈18	N/A

**Table 10:** Extended baseline comparison with safety metrics (30 runs).

Method	Adv. Profit	FPR (%)	Latency (×*T*)	Param Thrash	F1 Score
Baseline (Variance)	+145%	4.8	1.2T	–	0.75
Static Framework	+65%	4.1	1.1T	0.00	0.93
Thompson Sampling	+92%	6.2	1.3T	0.18	0.84
PID Controller	+78%	5.5	1.2T	0.22	0.87
EWMA Adaptive	+71%	5.1	1.2T	0.15	0.89
Contextual Bandit	+58%	4.9	1.3T	0.21	0.91
**DRL (Ours)**	−42%	**3.8**	1.1T	**0.09**	**0.95**

**Table 11:** Cross-DAA generalization: short window (W=144) to long window (W=2016).

Method	Same DAA	Cross DAA	Degradation
Static Framework	93% F1	87% F1	−6%
Thompson Sampling	84% F1	76% F1	−8%
**DRL Agent**	**95% F1**	**91% F1**	**−4%**

**Table 12:** Non-stationarity stress test results (30 runs, mean ± std).

Method	Rotating Pattern (Adv. Profit)	Stat Poisoning (Adv. Profit)
Static Framework	+88±9%	+112±11%
PID Controller	+72±8%	+95±10%
Mean/Std DRL	+35±7%	+68±9%
**MAD-Scaled DRL**	−28±6%	−15±5%

**Table 13:** Comparative AI model performance (30 runs, mean ± std).

AI Model	Precision	Recall	F1-Score
Supervised Classifier	99 ± 1%	65 ± 4%	0.78 ± 0.03
GAN Anomaly Detector	85 ± 3%	88 ± 3%	0.86 ± 0.02
**DRL Agent (Ours)**	**94 ± 2%**	**96 ± 2%**	**0.95 ± 0.02**

## Data Availability

The simulation environment is implemented in Python 3.10 using OpenAI Gym v0.26 for the RL interface, with the GRIDNET OS blockchain simulator providing high-fidelity PoW dynamics. The DRL agent uses PyTorch 2.0 with ONNX Runtime 1.15.1 export for deterministic deployment. Hyperparameters, random seeds for all 30 runs, and configuration files are documented for reproducibility. All simulation data, trained model weights, and source code are available from the corresponding author upon reasonable request. The reader may expect further information and discussions surrounding this research to be made available at https://mag.gridnet.org.
